# Obesity risk in young adults from the Jerusalem Perinatal Study (JPS): the contribution of polygenic risk and early life exposure

**DOI:** 10.1038/s41366-024-01505-7

**Published:** 2024-03-12

**Authors:** Hagit Hochner, Rachely Butterman, Ido Margaliot, Yechiel Friedlander, Michal Linial

**Affiliations:** 1 Braun School of Public Health, Hebrew University of Jerusalem, Jerusalem, Israel; 2 Department of Biological Chemistry, Institute of Life Sciences, The Hebrew University of Jerusalem, 91904 Jerusalem, Israel

**Keywords:** Genetics, Risk factors

## Abstract

**Background/Objectives:**

The effects of early life exposures on offspring life-course health are well established. This study assessed whether adding early socio-demographic and perinatal variables to a model based on polygenic risk score (PRS) improves prediction of obesity risk.

**Methods:**

We used the Jerusalem Perinatal study (JPS) with data at birth and body mass index (BMI) and waist circumference (WC) measured at age 32. The PRS was constructed using over 2.1M common SNPs identified in genome-wide association study (GWAS) for BMI. Linear and logistic models were applied in a stepwise approach. We first examined the associations between genetic variables and obesity-related phenotypes (e.g., BMI and WC). Secondly, socio-demographic variables were added and finally perinatal exposures, such as maternal pre-pregnancy BMI (mppBMI) and gestational weight gain (GWG) were added to the model. Improvement in prediction of each step was assessed using measures of model discrimination (area under the curve, AUC), net reclassification improvement (NRI) and integrated discrimination improvement (IDI).

**Results:**

One standard deviation (SD) change in PRS was associated with a significant increase in BMI (*β* = 1.40) and WC (*β* = 2.45). These associations were slightly attenuated (13.7–14.2%) with the addition of early life exposures to the model. Also, higher mppBMI was associated with increased offspring BMI (*β* = 0.39) and WC (*β* = 0.79) (*p* < 0.001). For obesity (BMI ≥ 30) prediction, the addition of early socio-demographic and perinatal exposures to the PRS model significantly increased AUC from 0.69 to 0.73. At an obesity risk threshold of 15%, the addition of early socio-demographic and perinatal exposures to the PRS model provided a significant improvement in reclassification of obesity (NRI, 0.147; 95% CI 0.068–0.225).

**Conclusions:**

Inclusion of early life exposures, such as mppBMI and maternal smoking, to a model based on PRS improves obesity risk prediction in an Israeli population-sample.

## Introduction

Obesity is a worldwide major health problem. A large body of literature has established a clear link between obesity with adverse health outcomes, including diabetes mellitus, coronary heart disease, stroke, heart failure and increased mortality [1, 2].

Risk factors for obesity have been studied extensively, and in general, they are divided into: demographic and socio-economic (e.g., education, and socioeconomic status) [3, 4]; environmental (e.g., unhealthy diet, smoking, and sedentary lifestyle), [5] and genetic factors [6, 7]. From a genetic perspective, the most important distinction is that between rare (monogenic) and common (multifactorial) obesity. Rare, high-risk genetic variations in roughly two dozen genes are known to cause monogenic obesity [8, 9]. However, for the vast majority of the population, obesity is polygenic, involving many common genetic variants with individually minor effects, collectively contributing to the majority of inherited susceptibility [9]. In the past 15 years, large-scale genome-wide association studies (GWAS) and meta-analyses have identified ~200 loci that are associated with adiposity traits across different populations worldwide [10–12]. However, single nucleotide polymorphisms (SNPs) identified in GWAS explain little of obesity variance [7, 13, 14]. Combining these SNPs into a polygenic risk score (PRS) has proven valuable, offering a quantifiable measure of inherited predisposition toward a trait, such as BMI [15]. Developing and validating obesity-specific PRS represents a breakthrough in personalized obesity and obesity-related disease risk prognosis [16]. Obesity-related PRS have been constructed in adults and children, displaying significant associations with numerous phenotypes and health outcomes such as circulatory, metabolic and other diseases, across diverse populations [17–19].

Beyond the aforementioned risk factors, current research indicates that fetal and early-life characteristics (like birth weight) play an important role in determining childhood and adulthood obesity. Maternal overnutrition, reflected in part by greater maternal pre-pregnancy body mass index (mppBMI) and gestational weight gain (GWG), has been consistently linked with offspring adiposity across their lifespan [20]. Other maternal attributes (e.g., educational attainment and maternal smoking during pregnancy) have also emerged as risk factors [21, 22].

Early intervention to prevent childhood and young adulthood obesity is key in attenuating the obesity epidemic [23]. While risk prediction based on a single genetic factor, such as PRS, has limited prognostic accuracy, combining multiple factors into a prediction model, enhances accuracy [24]. Additionally, studies indicate a significant impact of early-life social factors and maternal characteristics on the likelihood of adult obesity compared to similar factors measured during adulthood [25]. Thus, in this unique study prediction models of young-adulthood obesity were constructed using BMI-related PRS, early-life socio-demographic characteristics and perinatal traits. These attributes were collected through the Jerusalem Perinatal Study (JPS), alongside follow-up measurements of obesity-related traits at age 32. We employed both the conventional association approach, using measures such as explained variability (e.g., *R*^2^ statistic) and measures of performance of a prediction model (e.g., net reclassification improvement (NRI), and integrated discrimination improvement (IDI)) to determine if data collected during early life improve the prediction of adult obesity projected by PRS.

## Methods

### Study population

The JPS encompasses a subset of 17,003 births in Jerusalem from 1974 to 1976 [26]. Data consist of demographic and sociodemographic information, medical conditions of the mother during current and previous pregnancies, and offspring birth weight from birth certificates or maternity ward logbooks. In addition, mothers on the first or second day postpartum were interviewed regarding lifestyle and maternal health and provided data on gestational age, smoking status, height and pre-pregnancy weight, end of pregnancy weight and gynecological history.

The JPS Follow-up study conducted between 2007 and 2009 includes a sample of 2591 offspring with mean age 32 years (range 30–35 years) who were sampled from the original cohort [27] and 1440 were examined and interviewed (Supplementary Fig. 1). Sampling frame included singletons and term (gestational age ≥36 weeks) births without congenital malformations. We obtained a stratified sample of eligible individuals, where the strata were defined by mppBMI and birth weight (BW). Both low (≤2500 g) and high (≥4000 g) birth weight as well as mothers with overweight and obesity were over-sampled. Detailed information on data collection has been previously described [27]. Height, weight, waist measurements and fasting blood samples were collected following standard protocols.

This study was approved by the Institutional Review Board of the Hadassah-Hebrew University Medical Center and all participants provided informed consent. Data are stored in the European Genome-phenome Archive, https://ega-archive.org/ accession number EGAS00001004075.

### Study variables

The primary outcome examined was offspring’s BMI and waist circumference (WC), treated as continuous variables. Additionally, this study also examined two outcomes: (1) obesity, defined as BMI ≥30, and (2) central obesity, defined as WC >8th decile of the gender specific WC distribution [28, 29].

In addition, the following explanatory variables were examined: early life socioeconomic status (SES) based on father’s occupation (grouped into six categories: 1 to 6, for lower to upper class, respectively) which was found to be similar and well correlated with other Israeli social class scales [4, 26], mother’s years of education (continuous), parental age at offspring’s birth (continuous), birth weight (BW) (gr, continuous), gestational age (weeks, continuous), mppBMI (kg/m^2^, continuous) and GWG (kg, continuous), and maternal smoking during pregnancy (categorical: current smoker (including those who quit during the current pregnancy) vs. never smoked or smoked in the past and quit before the this pregnancy. Father’s occupation was selected as an indicator of early life SES, rather than mother’s occupation, as approximately half of the women in this cohort were not employed outside the home during childbirth [26].

### Genotyping and quality control

Genomic DNA was extracted using the salting-out method, then genotyping was carried out using the Affymetrix Biobank array (comprising of 587,021 variants). We used standard quality control criteria for individual samples and genetic variants [30, 31]. Out of 2790 DNA samples of both parents and offspring (950 triads), 68 samples had poor DNA quality and 138 samples did not pass the QC due to high rate of missing information and/or high rate of heterozygosity. Overall, we were left with 2593 samples, 872 from offspring. We started the SNPs QC for 587,021 SNPs with high variant calling rates. The missingness test had filtered out 22,151 SNPs, 166,313 SNPs were defined as monomorphic (frequency <0.00001) and in addition, we filtered out 142 SNPs with more than 100 Mendelian errors. Thus, a total of 398,491 validated SNPs were available for the analysis.

Imputation was done using a combined panel of the 1000 genomes [32] and The Ashkenazi Genome Consortium (*n* = 128) as ref. [33]. Imputed variants with MAF ≥ 1% and quality score ≥0.9 are retained (hard calls), generating a high-quality genetic dataset with nearly 7M genetic variants per individual available for subsequent analyses.

### PRS calculation

PRS was computed for offspring genotype data using parameters derived from BMI-related GWAS. The association study employed BMI summary statistics from various European ancestry populations, encompassing 339,224 individuals from 125 studies, either with GWAS results or with results from Metabochip studies [18, 34], and a LD reference panel of 503 European samples from 1000 Genomes Phase 3, Ver 5 [32]. The results from multiple PRS that we have calculated showed that using only 10 to 100 SNPs which were found significant by the original GWAS was insufficient to create a reliable PRS. In fact, as many as 500k to 700k SNPs were needed to reach 90 and 95% of the maximal achieved PRS-BMI correlation. The majority of the variants (up to 2 million) contributed positively to this correlation and therefore for the current analyses we used a panel which consisted all 2,100,302 common variants (MAF ≥ 1%). The PRS was established by the LDPred computational algorithm, a Bayesian method that calculates a posterior mean effect for all variants using external weights, then shrinks them based on linkage disequilibrium [35].

Principle component analysis (PCA) was applied on genotyped samples of both parents and offspring (*n* = 2575) along with Jewish reference panel (*n* = 174) using PC-Air [36]. Rather than assigning offspring to a single ethnicity, the first five PCs were included as covariates in regression models to account for variations in ancestry within the Jewish population in Israel.

### Statistical analyses

Linear regression models were used to explore the associations of the genetic group (i.e., PRS and 5 PCs), socio-demographic group (i.e., parental age at offspring birth and SES at birth) and perinatal characteristics group (i.e., Gestational week, mppBMI, GWG, BW and parental smoking during pregnancy) independent of each other, with BMI and WC. Four sets of models were constructed; Model 1 included sex as covariate, Model 2 included PRS adjusted for ethnicity (PCs 1–5) and sex, Model 3 incorporated socio-demographic characteristics, and Model 4 incorporated perinatal characteristics. Since these models were nested it enabled an assessment of each variable group’s contribution to explained BMI and WC variability. For categorical outcomes (obesity and central obesity), we used logistic regression analyses. Since the analyses of our dataset used probability weights, the likelihood-based tests may not be valid. An alternative method is comparing predicted probabilities from each model to assess their performance.

The area under the curve (AUC) was applied in evaluating the effectiveness of various diagnostic-models in separating between subjects with and without obesity [37]. Internal validity of the prediction model was assessed by 10-fold cross-validation.

The net reclassification improvement (NRI) index [38], was used to compare the saturated model (Model 4) and the restricted models (Model 2 and Model 3) for their ability to classify each subject either with or without obesity. The overall NRI which is the sum of improvement in reclassification of each obesity group (predicted risk <15 or ≥15%) was examined by *χ*^2^ values. A category-free version of the NRI was also presented [39] to ascertain risk increases to any extent for subjects with obesity under the saturated model (Model 4) compared to the restricted models, and similarly whether risk decreases to any degree for subjects without obesity.

## Results

### Population characteristics

Table 1 presents maternal and offspring birth characteristics, offspring PRS and obesity-related traits at age 32. Among mothers (around age 27.5), mppBMI was 23.8 kg/m^2^ and GWG was 11.3 kg. Mean offspring BMI at age 32 was 26.7, with greater WC among males than females. Overweight prevalence (BMI > 25) was higher in males (41.1%) than females (25.9%).

**Table 1:** Descriptive characteristics^a^ of study participants by sex groups.

Variable	Females (*n* = 401)	Males (*n* = 438)	Total (*n* = 839)
PRS^b^	0.051 (1.03)	−0.03 (0.97)	0.01 (1.00)
Mother age at birth, years	27.6 (5.4)	27.4 (5.3)	27.5 (5.3)
Father age at birth, years	31.0 (6.4)	31.2 (6.39)	31.1 (6.3)
Socioeconomic status at birth %			
High	37.9	44.3	41.2
Medium	41.4	32.9	36.6
Low	20.7	22.8	21.8
Gestational week, weeks	39.9 (1.5)	40.0 (1.4)	40.0 (1.5)
Maternal pre-pregnancy BMI, kg/m^2^	24.2 (4.0)	23.6 (3.5)	23.8 (3.7)
Gestational weight gain, kg	10.9 (4.7)	11.6 (4.5)	11.3 (4.6)
Birth weight, gr	3278 (591)	3553 (593)	3421 (608)
Birth weight %			
Low (≤2500 g)	14.5	7.8	11.0
Medium (2501– 4000 g)	71.3	66.0	68.5
High (>4000 g)	14.2	26.3	20.5
Parental smoking during pregnancy^c^ %	47.6	43.1	45.3
BMI, kg/m^2^	25.6 (5.2)	26.8 (4.8)	26.7 (5.0)
Waist circumference, cm	80.8 (12.1)	91.1 (12.2)	86.2 (13.2)
Adiposity %			
Normal (BMI < 25.0)	55.8	38.8	47.0
Overweight (BMI 25.0–29.9)	25.9	41.1	33.8
Obesity (BMI ≥ 30.0)	18.2	20.1	19.29

^a^Values are expressed as percent or mean (SD).

^b^Polygenic risk score.

^c^Either mother and/or father.

### Risk factor association

#### BMI and obesity

Linear regression models’ outcomes are displayed in Table 2a, examining the genetic, socio-demographic, and perinatal factors associated with offspring BMI. Model 2, showed a significant BMI increase (1.4 kg/m²) per one standard deviation increase in PRS. Notably, this PRS association is independent of sex and the first 5 PCs.

**Table 2:** Association analysis of obesity-related traits with groups of risk factors.

		Model 1		Model 2		Model 3		Model 4	
Group	Variable	*β*	(95% CI)	*β*	(95% CI)	*β*	(95% CI)	*β*	(95% CI)
a. Parameter estimates from linear regression models of BMI as predicted by various groups of risk factors									
1	Sex	1.74***	0.86 2.61	1.68***	0.85 2.51	1.60***	0.77 2.44	1.70***	0.87 2.53
2	PRS			1.40***	0.97 1.83	1.41***	0.99 1.84	1.21***	0.77 1.65
	PC1			0.70**	0.23 1.17	0.89***	0.36 1.41	0.73**	0.20 1. 27
	PC2			0.20	−0.20 0.61	0.13	−0.29 0.56	0.06	−0.47 0.36
	PC3			−0.04	−0.56 0.48	−0.01	−0.52 0.51	0.05	−0.56 0.46
	PC4			0.30	−0.26 0.86	0.35	−0.21 0.90	0.37	−0.91 0.17
	PC5			0.36*	0.06 0.66	0.37*	0.05 0.69	0.26	−0.60 0.08
3	Paternal SES					0.19	−0.10 0.48	0.11	−0.39 0.17
	Maternal age					−0.07	−0.19 0.06	−0.08	−0.04 0.20
	Paternal age					0.03	−0.08 0.14	0.02	−0.12 0.09
4	Birth weight							0.19	−0.84 0.46
	Gestational week							−0.23	−0.09 0.56
	ppmBMI							0.39***	0.29 0.50
	Parental smoking							0.60	−1.44 0.25
	GWG							0.07	−0.17 0.02
	*R*^2^	0.0346		0.1271		0.1329		0.1964	
b. Parameter estimates from linear regression models of waist circumference as predicted by various groups of risk factors									
1	Sex	11.33***	9.1413.52	11.22***	9.06 13.39	11.05***	8.89 13.21	11.29***	9.15 13.43
2	PRS			2.45***	1.27 3.64	2.48***	1.31 3.65	2.14***	0.90 3.38
	PC1			1.75**	0.54 2.97	2.15**	0.82 3.48	1.86**	0.51 3.21
	PC2			−0.08	−1.16 1.00	−0.23	−1.36 0.89	−0.41	−1.49 0.67
	PC3			−0.1	−1.29 1.10	−0.03	−1.23 1.17	−0.04	−1.22 1.15
	PC4			0.47	−1.29 1.10	0.58	−0.68 1.83	0.61	−0.60 1.82
	PC5			0.81*	0.01 1.62	0.82*	−0.01 1.65	0.57	−0.31 1.46
3	Paternal SES					0.4	−0.28 1.08	0.21	−0.46 0.88
	Maternal age					−0.15	−0.49 0.18	−0.19	−0.51 0.14
	Paternal age					0.06	−0.23 0.36	0.04	−0.26 0.33
4	Birth weight							1.4	−0.27 3.07
	Gestational week							−0.66	−1.50 0.17
	ppmBMI							0.79***	0.52 1.07
	Parental smoking							2.33*	0.11 4.54
	GWG							0.03	−0.22 0.29
	*R*^2^	0.1982		0.2412		0.2452		0.2877	

*PRS* polygenic risk score, *PC* principal component, *SES* socioeconomic status, *ppmBMI* pre-pregnancy maternal BMI, *GWG* gestational weight gain.

*0.01 < *p* ≤ 0.05; **0.001 < *p* ≤ 0.01; ***p ≤ 0.001.

Socio-demographic factors contributed minimally to the explained variability of offspring BMI (Δ*R*^2^ = 0.0058; *p* = 0.183; Model 3).

Upon inclusion of perinatal variables into the model, the explained variability of offspring BMI notably increased from *R*^2^ of 0.133 (in Model 3) to 0.196 (in Model 4), *F*_5, 823_ = 12.9; *p* ≤ 0.001). Each SD change in mppBMI was associated with 0.39 kg/m² increase in offspring BMI (*p* ≤ 0.001). The association between PRS and offspring BMI was slightly attenuated (though remained significant) with the addition of perinatal variables into the model.

**Fig. 1:**
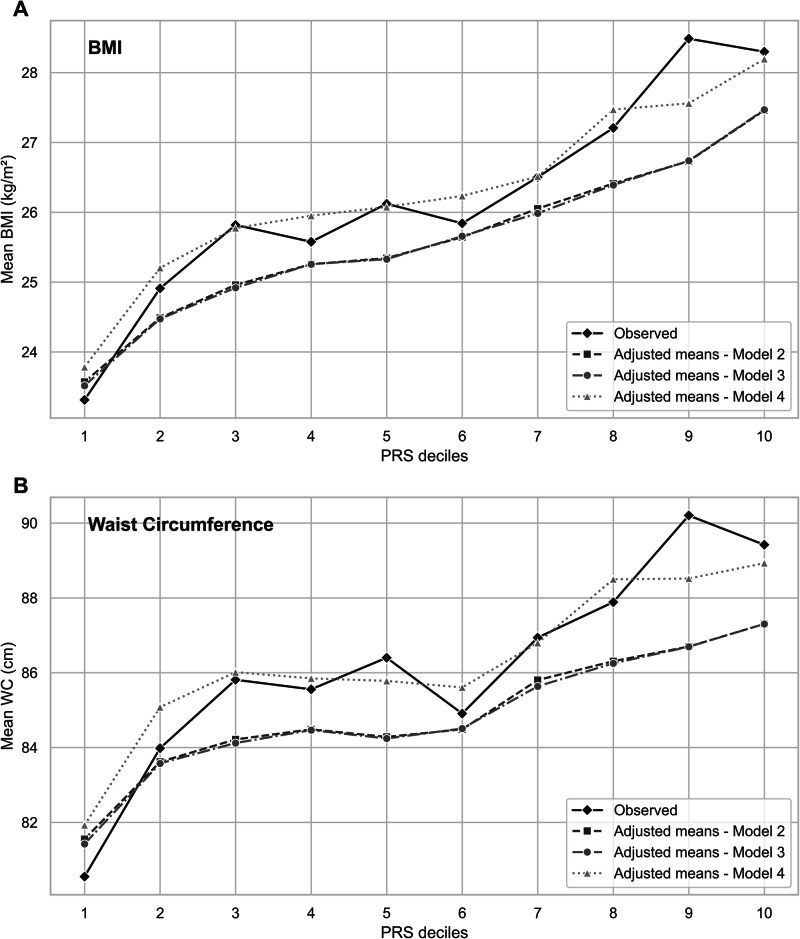
Observed and predicted means partitioned by PRS deciles. **A** shows observed and predicted BMI adjusted means generated from Model 2 (Genetic), Model 3 (Genetic + Socio-demographic), and Model 4 (Genetic + Socio-demographic + Perinatal). Similarly, **B** shows observed and predicted waist circumference (WC) adjusted means estimated from Model 2 (Genetic), Model 3 (Genetic + Socio-demographic), and Model 4 (Genetic + Socio-demographic + Perinatal).

Figure 1A illustrates observed and adjusted offspring BMI means across PRS deciles, showing substantial shifts between the 3 lower and 3 upper deciles. For example, mean observed and predicted BMI levels for offspring located at the upper PRS decile were nearly 5 kg/m² higher compared with mean levels of BMI in the lowest decile of PRS.

Additionally, logistic regression models were used to examine these associations with dichotomous measures of obesity (BMI ≥ 30). Supplementary Table 1a reveals a significant PRS-obesity association (adjusted odds ratio [OR] per SD increment, 2.08; 95% CI, 1.56–2.77). In Model 4, OR changes for genetic variables were minor. Yet, both ppmBMI (adjusted OR per SD increment, 1.22; 95% CI, 1.14–1.30) and GWG (adjusted OR per SD increment, 1.08; 95% CI, 1.01–1.15) were significantly associated with obesity.

#### Central obesity

Similar to BMI, the association between PRS deciles and WC mean levels is clearly demonstrated across the lowest and the highest deciles of PRS (Fig. 1B).

Table 2b presents results of linear regression models examining the associations with offspring WC. Sex strongly associated with offspring WC and explained a substantial proportion of its variability (*R*^2^ = 0.19). Incorporating PRS, the change in explained WC variability was modest, yet significant, (Δ*R*^2^ = 0.043), and the independent contribution of socio-demographic variables was negligible (Δ*R*^2^ = 0.004; *p* = 0.282).

Incorporating perinatal variables in Model 4 (Table 2b), significantly increased WC explained variance (Δ*R*^2^ = 0.0425, *F*_5, 823_ = 9.821; *p* ≤ 0.001). Maternal smoking and mppBMI, adjusted for other perinatal and socio-demographic characteristics and for genetic factors, were positively associated with offspring WC. The association between PRS and WC was attenuated (though remained significant) when perinatal variables were included in the model.

Finally, logistic regression models applied to categorical measures of central obesity (WC > 8th decile), confirmed that similar to the association with obesity, both PRS and ppmBMI were significantly associated with central obesity (Supplementary Table 1b, Model 4).

### Risk prediction—discrimination and reclassification

#### Obesity

**Fig. 2:**
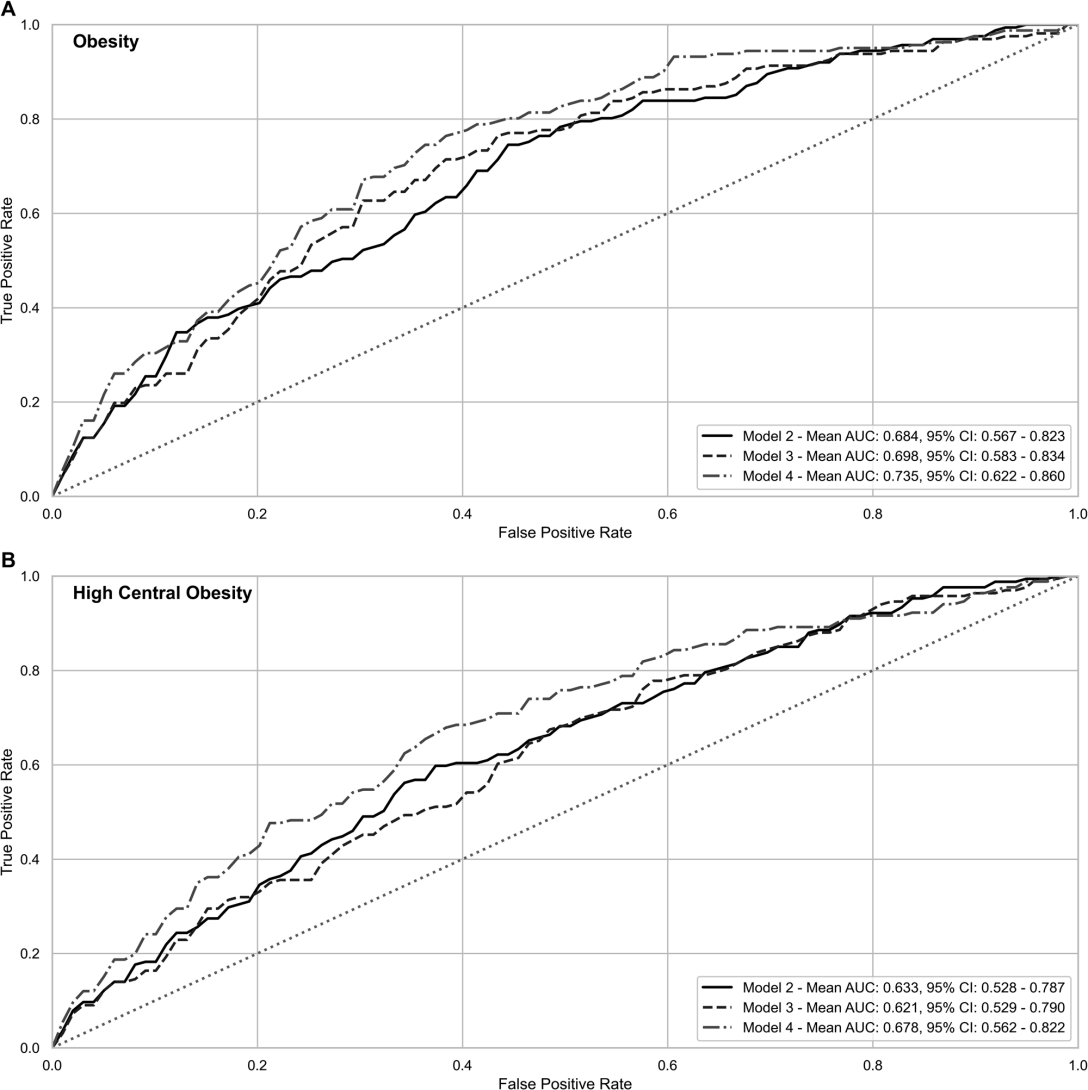
Discrimination between subjects with and without obesity, estimated by parameters of Model 2 (Genetic), Model 3 (Genetic + Socio-demographic), and Model 4 (Genetic + Socio-demographic + Perinatal). Mean AUC (95% confidence interval) are presented where AUCs are based on 10-fold cross-validation for obesity (BMI ≥ 30 kg/m^2^) (**A**) and high central obesity (WC > 8th decile) (**B**).

Our data showed that a risk model based on PRS had a sensitivity of 0.80 and specificity of 0.54 to predict young adult obesity (BMI ≥ 30) and the positive predictive value (PPV) and negative predictive value (NPV) of PRS were 0.27 and 0.91, respectively. Overall, the potential discrimination for predicting obesity in offspring increased when the perinatal variables were included in the model (sensitivity = 0.84, specificity = 0.60, PPV = 0.33 and NPV = 0.94). Figure 2A presents AUCs that estimate the discrimination between subjects with and without obesity (BMI ≥ 30) across the combination of risk predictors. The 10-fold average AUC increased modestly from 0.68 (Model 2—genetic) to 0.70 (Model 3—genetic + socio-demographic) and considerably to 0.74 when genetic + socio-demographic + perinatal variables were included in Model 4.

Next, we determined calibration by comparing observed and expected frequency of obesity-related outcomes from models without (Model 2) and with (Model 4) socio-demographic and perinatal variables. Model 2 categorized 43.9% of the sample as low risk (predicted risk ≤15%) and 56.1% as high risk (predicted risk >15%) (Table 3). Observed proportion of subjects without and with obesity in these 2 groups were 91.0% (NPV) and 27.2% (PPV), respectively. Adding socio-demographic and perinatal variables to the model (Model 4), adjusted this to 51.4% and 48.6% as low and high-risk subjects, respectively, with improved accuracy (observed proportion of subjects without and with obesity of 94.0% and 33.1%, respectively). When adding to Model 2 the socio-demographic variables (Model 3) the NRI was somewhat improved (NRI, 0.073; 95% CI, 0.0291–0.1162), however when both the socio-demographic and perinatal variables were added to Model 2, the risk categories significantly improved reclassification accuracy in obesity (NRI, 0.147; 95% CI, 0.068–0.225), where net proportion of correct reclassification for subjects with obesity was 7/161 = 0.044 and 70/678 = 0.103 in subjects without obesity (Table 4).

**Table 3:** Risk of obesity predicted by logistic models without and with perinatal characteristics: the Jerusalem Perinatal Study.

Predicted risk by model with perinatal variables						
		<15%	≥15%	Overall	Reclassified as higher risk	Reclassified as lower risk
Predicted risk by model without perinatal variables						
Obesity						
<15%						
	No. of subjects with obesity	14	19	33	19 (True)	NA
	No. of subjects without obesity	282	53	335	53 (False)	NA
	Total no. of subjects	296	72	368		
≥15%						
	No. of subjects with obesity	12	116	128	NA	12 (False)
	No. of subjects without obesity	123	220	343	NA	123 (True)
	Total no. of subjects	135	336	471		
Overall						
	No. of subjects with obesity	26	135	161		
	No. of subjects without obesity	405	273	678		
	Total no. of subjects	431	408	839		
Central obesity						
<15%						
	No. of subjects with obesity	19	9	28	9 (True)	NA
	No. of subjects without obesity	235	47	282	47 (False)	NA
	Total no. of subjects	254	56	310		
≥15%						
	No. of subjects with obesity	10	128	138	NA	10 (False)
	No. of subjects without obesity	116	275	391	NA	116 (True)
	Total no. of subjects	126	385	529		
Overall						
	No. of subjects with obesity	29	137	166		
	No. of subjects without obesity	351	322	673		
	Total no. of subjects	380	459	839

**Table 4:** Comparison of reclassification and discrimination improvement models for obesity-related variables.

	Prediction improvement (95% CI)		
Variable	Model 2 vs. Model 3	Model 3 vs. Model 4	Model 2 vs. Model 4
Obesity—NRI^a^	0.073 (0.0291 0.1162)	0.074 (0.0036 0.1445)	0.147 (0.0689 0.2250)
Obesity—IDI^b^	0.013 (0.0043 0.0218)	0.049 (0.0993 0.0680)	0.062 (0.0407 0.0833)
Central obesity—NRI^a^	0.015 (−0.0293 0.0592)	0.082 (0.0204 0.1435)	0.097 (0.0334 0.1601)
Central obesity—IDI^b^	0.005 (−0.0001 0.0107)	0.044 (0.0271 0.0609)	0.049 (0.0307 0.0672)

^a^Net reclassification index.

^b^Integrated discrimination improvement.

Supplementary Fig. 2a depicts predicted probabilities based on the different models for subjects with and without obesity. These results show that 72.7% (117/161) of subjects with obesity (BMI ≥ 30) had a predicted risk based on model 4 higher than the predicted risk based on model 2, i.e., a more accurate risk estimate (Panel E). Conversely, 27.3% had a higher predicted risk based on model 2 (Panel E). The net proportion of risk estimates with higher accuracy (obese NRI (>0)) hence was 45.4%. In addition, we note that 54.9% of subjects without obesity had a lower prediction based on model 4, while 45.1% had a higher prediction based on Model 4 (non-obese NRI (>0) = 9.5%; Panel F). The sum of the two components of NRI (>0) equals 54.8%. The integrated discrimination improvement (IDI) also showed a significant improvement favoring the model with PRS and perinatal variables as compared to the model without perinatal characteristics (0.062%; 95% CI 0.040–0.083; Table 4). The improvement in IDI was nearly 5-times greater compared to the improvement observed when solely integrating socio-demographic variables into the PRS model (Model 3 vs. Model 2) (0.013%; 95% CI 0.0043–0.0218, Table 4).

#### Central obesity

Similarly, Fig. 2B presents AUC that estimate the discrimination between subjects with and without high central obesity (WC > 8th decile) across the combination of risk predictors. The 10-fold average AUC increased only when the perinatal variables were included in the model from 0.62–0.63 (Models 2 and 3) to 0.68 (Model 4).

Regarding central obesity, Model 2 categorized 36.9% of the sample as low-risk and 63.1% as high-risk (Table 3). In these groups, 91.0% without and 26.1% with central obesity were observed. Model 4, compared to Model 2, placed more individuals in low-risk (45.3%) and fewer in high-risk (54.7%) for central obesity. The observed proportion of subjects without and with central obesity in these 2 groups were 92.4% and 29.8%, respectively.

Analyses suggested a better net reclassification of central obesity using Model 4 (NRI, 0.097; 95% CI, 0.034–0.159), with a similar proportion of correct reclassification of −0.006 in subjects with central obesity and an improved reclassification of 0.097 in subjects without central obesity (Table 4). There was no significant improvement in NRI when only socio-demographic variables were added to the genetic model (0.015 95% CI −0.0293 to 0.0592).

Similarly, Supplementary Fig. 2b (Panel E and Panel F) indicated that the sum of the two components of the continuous NRI (NRI > 0) was about 40.0%. Yet, the better risk prediction based on Model 4, was limited to subjects with central obesity. IDI was also improved (0.049%; 95% CI 0.0307 to 0.0672), favoring the combination of PRS, socio-demographic and Perinatal model (Table 4). This improvement in IDI was almost 10-fold compared to improvement derived from adding only the socio-demographic variables to the PRS model (Model 3 vs. Model 2) (0.005%; 95% CI −0.0001 to 0.0107, Table 4).

## Discussion

In this work our objective was to investigate the impact of PRS for BMI, socio-demographic characteristics, and perinatal exposures on the risk of obesity among young adults. To achieve this, we employed two methodologies. Initially, we modeled the associations of these factors with obesity-related traits and assessed the contribution of each set of factors to the explained variability of obesity traits. Subsequently, we developed prediction models and assessed their effectiveness and performance.

Results of the association analyses revealed significant findings regarding the relationship between genetic factors and obesity-related traits in young adults. We measured the PRS for BMI using multiple loci from SNPs derived from GWAS of BMI in the UK Biobank [18, 34, 40]. PRS displayed a significant association with obesity and central obesity, albeit contributing modestly to the explained variability (4–9%). The correlation observed of BMI-PRS (with sex and 5 PCs included in the model) with BMI (*r* = 0.3) and WC (*r* = 0.2) were comparable to those reported in previous studies. For example, a large study involving middle-aged subjects (*n* = 288,016, mean age = 57) from the UK Biobank, demonstrated a PRS approximating a normal distribution which was significantly associated with BMI values (*r* = 0.29) [18]. Similar to our findings, in another large study based on young women from the Nurses’ Health Study and young men from the Health Professionals Follow-Up Study, each 10-allele increment in BMI-PRS was associated with an average BMI gain of 0.54 kg/m^2^ in women and 0.20 kg/m^2^ in men [41]. In a prospective longitudinal study of participants in the Dunedin birth cohort, children with higher PRS had higher BMIs at every age assessed, from age 3 through age 38 years [17]. At ages 30–38 the PRS correlation with BMI (*r* = 0.13–0.14) and with WC (*r* = 0.11–0.12) were statistically significant and similar, yet, these correlations were somewhat lower compared to our results.

Additionally, we show independent associations of PRS and perinatal exposures, such as maternal pregnancy-related body size, with offspring BMI and central obesity. Interestingly, associations between PRS and offspring BMI and WC were marginally attenuated (14.2% and 13.7%, respectively) with the inclusion of perinatal variables. It is plausible that common genetic variation contributed to both maternal and offspring phenotype levels, leading to a slight attenuation in the association between PRS and offspring obesity-related traits when perinatal variables were added to the model. Consistent with our findings, within the Dunedin birth cohort, PRS was associated with offspring BMI independently of family history of obesity in their parents [17]. Thus, PRS may contain additional information about children’s risk for obesity beyond their parental history of obesity. However, while the PRS was associated with offspring obesity at young adulthood independently of birth weight, adiposity rebound tended to mediate a large portion of PRS effect on young adulthood obesity [17].

Similar to other studies in Caucasian and non-Caucasian populations [42] we have demonstrated a distinct variation in WC levels between sexes. This discrepancy emerges in early life and is attributed to differences in body composition, fat distribution and sex hormone profiles. As opposed to this sex difference in WC and the observed effects of the perinatal variables, we show that parental ages and socio-economic status (SES) were not associated with offspring’s BMI and WC at age 32 and their inclusion in the model did not change the regression coefficients for PRS. Our findings contradict those observed in a birth cohort of White Western European subjects, in which both lower SES and high PRS were associated with increased pre-adolescence BMI [43]. The effect of SES was largely independent of the effect of the PRS and the inclusion of both variables in the model did not substantially alter the effect sizes observed when each variable was included alone. A more complex picture was observed in a study spanning three generations of birth cohorts in Finland, exploring associations of early-life social disadvantage with later-life BMI [25]. In the older two cohorts there was either no association between social disadvantage and BMI or an association independent of PRS (albeit slightly attenuated). However, within the youngest cohort, similar to our findings, the association between early social disadvantage and later life BMI was fully attenuated after adjusting for PRS. The authors suggested genetic selection and assortative mating may partly explain the social selection observed in the youngest age group. In our study, we suggest that the full attenuation of the socio-demographic factors when PRS and PCs are included could be also attributed to the confounding effect of ancestry, represented by the PCs. In the Jewish Israeli population ethnicity is strongly associated with socio-demographic characteristics [44] and thus the presence of PCs in the model likely diminishes their effect on BMI.

The second approach we took to address our study aims was to construct prediction models for obesity (BMI ≥ 30) and central obesity (WC > 8th decile). We showed that the capacity of the PRS model to discriminate between individuals with and without obesity was AUC = 0.69, and this was improved when perinatal variables were additionally included (AUC = 0.73). We also demonstrated this improvement using other measures of prediction performance (i.e., NRI and IDI). The results for the risk of central obesity paralleled those of overall obesity, yet the models provided slightly less accurate estimates compared to models based on BMI.

Several studies have examined obesity risk prediction models based on PRS. Interestingly, regardless of the number of loci included in the score, the models’ discrimination capacity for obesity ranged from AUC of 0.57–0.61, in line with our results [7, 45, 46]. Others have examined whether obesity risk prediction is improved with addition of risk factors to the model, above and beyond PRS. For example, in a Danish study, the AUC for a genetic model (PRS only) was 0.58 and after the inclusion of non-genetic obesity risk factors (e.g., education, diet, smoking, and physical activity) the AUC increased to 0.69 [47]. Compared to our findings, the contribution of risk factors to obesity prediction was even more substantial, yet overall performance of the saturated models in both studies are equivalent.

Furthermore, using a 15% threshold, we showed increased discrimination ability for predicting offspring’s obesity when the perinatal variables were also included in the model (sensitivity = 0.84, specificity = 0.60, PPV = 0.33 and NPV = 0.94). A previous study showed that parental obesity status at age 6–9 years had a sensitivity, specificity, PPV and NPV of 0.58, 0.70, 0.27 and 0.90 respectively, predict adult obesity [7, 48]. The resemblance to our results might imply that family history encompasses both genetic and non-genetic components which contribute to obesity prediction in offspring. However, the observed differences in results, particularly the slightly heightened sensitivity and reduced specificity in our study, could be partially explained by the relatively older age in which maternal pre-pregnancy BMI was assessed (i.e., adulthood in our study vs. childhood). It has been shown that the predictive sensitivity of parental obesity for anticipating the risk of adult obesity in offspring may rise with the age of parental obesity assessment, while specificity tends to diminish with parental age [7].

Inspection of the proportion of correct reclassification among subjects who are either with or without obesity highlights the strengths and weaknesses of the combined PRS-perinatal strategy and provides insights on its implications. Overall, 25% of total subjects were reclassified, of which 17% were reclassified correctly, the majority (15%) being without obesity. The improved performance among subjects without obesity is also reflected by the higher NRI for subjects without obesity (0.103) compared to subjects with obesity (0.044), totaling the reported overall NRI of 0.147. This is also supported by the high NPV (0.94). These figures provide confidence that only a limited number of offspring predicted to be at low risk for obesity in adulthood by the combined genetic-perinatal model will eventually become overweight or obese. However, prediction of high obesity risk is less accurate and thus a larger number of those identified will in fact not develop obesity in the future. Nonetheless, the advantage of PRS and early life exposures is that they can be assessed at young ages long before adverse outcomes are manifested. If these markers are indeed used for early screening, identified high-risk individuals could truly benefit from early lifestyle modifications (e.g., healthy diet and increased physical activity). Importantly, such behavioral and/or environmental interventions to tackle obesity are in fact beneficial, even if applied among low-risk individuals.

The major strength of our study is the combination of high-quality detailed records of prenatal and perinatal characteristics with a comprehensive long-term follow-up data.

There are also several limitations to our study. First, it includes only a sample of offspring from the original 1974–1976 JPS cohort who were invited to participate in the follow-up study. However, using a stratified sampling approach and over-sampling the ends of the distribution ensured that offspring with a full range of mppBMI and birth weight were included in our study. Second, the PRS explained only a modest percent of the variation for BMI. This is in agreement with other studies, and expected with the current methodology used [18, 25]. Third, both mppBMI and GWG were reported by mothers in interviews conducted by nurses while hospitalized after delivery, whereas, verification from clinical records was not available. Nevertheless, we have demonstrated associations between reported maternal attributes and BMI more than 30 years later, as well as with long-term clinical outcomes in mothers [49], which lend support to the validity of the data. Additionally, in our study mothers were interviewed within days after delivery, in accordance of the reports on the validity and reproducibility of maternal recollection of pre-pregnancy weight [50, 51]. Even if reporting error was present, it is most likely that this non-differential misclassification resulted in an underestimation in our findings.

## Conclusions

This study underscores the strong relationship between BMI and the risk of obesity in early adulthood with PRS and with maternal excess weight before pregnancy. In addition, our prediction models demonstrate that utilizing PRS while combining perinatal exposures enhances the ability to quantify future obesity risk in young adults. These findings offer impetus to progress toward the subsequent evaluation phase, in which PRS and early-life exposures could be utilized to target young individuals at higher obesity risk who could benefit from early preventive interventions.

## Summary

### What was known before?

- Studies demonstrate associations between obesity-related phenotypes and subsequent risk of various diseases.
- Genetic factors explored through polygenic risk score (PRS) provides valid association estimates with obesity throughout adolescence and adulthood.
- Evidence also suggests that early life exposures, such as maternal pre-pregnancy body mass index (mppBMI) and gestational weight gain (GWG), are associated with adult offspring adiposity.

### What this study adds?

- Our analyses show that both genetic factors and early life exposures were significantly and independently associated with obesity-related outcomes.
- Our study also demonstrates that inclusion of early life exposures improved the obesity risk-prediction model relative to a model that solely considered genetics factors.

### How might these results change the direction of research or the focus of clinical practice?

- Our prediction models demonstrate that combining PRS with early life exposures results in improved capacity to estimate future obesity risk in young adults.
- These findings offer impetus to progress toward the subsequent evaluation phase, in which PRS and early-life exposures could be utilized to target young individuals at higher obesity risk who could benefit from early preventive interventions.

### Supplementary information

Supplementary materials

## Data Availability

JPS data reported in this paper can be found in the European Genome-phenome Archive (EGA), https://ega-archive.org/, under accession number EGAS00001004075. Open access funding provided by Hebrew University of Jerusalem.
