# Supplementary material for: Obesity risk in young adults from the Jerusalem Perinatal Study (JPS): the contribution of polygenic risk and early life exposure
Source: Int J Obes (Lond). 2024 Mar 12;48(7):954–63. doi: 10.1038/s41366-024-01505-7 (PMC11216986; doi:10.1038/s41366-024-01505-7)

Supplement Table 1a. Parameter Estimates from logistic models of **o**besity as predicted by various groups of risk factors

		Model 1		Model 2		Model 3		Model 4	
Group	Variable	OR	(95% CI)	OR	(95% CI)	OR	(95% CI)	OR	(95% CI)
1	Sex	1.28	0.76 2.17	1.30	0.74 2.27	1.22	0.69 2.17	1.21	0.66 2.24
	PRS			2.08***	1.56 2.77	2.11***	1.57 2.83	1.99***	1.43 2.76
	PC1			1.56**	1.14 2.13	1.87***	1.28 2.72	1.84**	1.21 2.78
2	PC2			1.06	0.80 1.40	0.98	0.72 1.33	0.92	0.67 1.26
	PC3			0.93	0.68 1.26	0.94	0.68 1.30	0.97	0.69 1.36
	PC4			1.21	0.89 1.66	1.26	0.91 1.77	1.34	0.97 1.84
	PC5			1.39*	1.04 1.85	1.37*	1.03 1.82	1.39*	1.02 1.88
	Paternal SES					1.19	0.99 1.44	1.13	0.93 1.37
3	Maternal age					0.96	0.88 1.05	0.95	0.86 1.04
	Paternal age					0.98	0.90 1.06	0.97	0.89 1.06
	Birth weight							0.86	0.55 1.36
	Gestational week							0.93	0.75 1.16
4	ppmBMI							1.22***	1.14 1.30
	Parental smoking							1.59	0.86 2.95
	GWG							1.08*	1.01 1.15
	-2lnL	2,270.10		2,097.13		2,045.97		1,899.76	

PRS - polygenic risk score, PC – principal component SES – Socioeconomic status; ppmBMI – pre-pregnancy maternal BMI; GWG – gestational weight gain

* p≤0.05; ** p≤0.01; *** p≤0.001

Supplement Table 1b. Parameter Estimates from logistic models of central obesity as predicted by various groups of risk factors									
		Model 1		Model 2		Model 3		Model 4	
Group	Variable	OR	(95% CI)	OR	(95% CI)	OR	(95% CI)	OR	(95% CI)
1	Sex	1.30	0.78 2.16	1.29	0.76 2.19	1.20	0.69 2.08	1.24	0.70 2.21
	PRS			1.67***	1.29 2.15	1.67***	1.30 2.16	1.63***	1.24 2.15
	PC1			1.50**	1.11 2.03	1.72**	1.21 2.45	1.66**	1.15 2.40
2	PC2			1.11	0.84 1.45	1.04	0.79 1.37	1.01	0.77 1.33
	PC3			0.99	0.75 1.30	1.01	0.75 1.35	1.00	0.74 1.35
	PC4			1.26	0.95 1.67	1.31	0.97 1.76	1.33	0.99 1.80
	PC5			1.44*	0.95 1.67	1.43*	1.07 1.91	1.42*	1.05 1.92
	Paternal SES					1.12	0.96 1.30	1.07	0.92 1.24
3	Maternal age					0.94	0.85 1.05	0.94	0.85 1.04
	Paternal age					1.00	0.91 1.10	0.99	0.90 1.09
	Birth weight							1.21	0.78 1.86
	Gestational week							0.88	0.71 1.08
4	ppmBMI							1.16***	1.08 1.23
	Parental smoking							1.75	0.97 3.14
	GWG							0.99	0.93 1.06
	-2lnL	2,429.21		2,299.87		2,260.80		2,160.59	

PRS - polygenic risk score, PC – principal component SES – Socioeconomic status; ppmBMI – pre-pregnancy maternal BMI; GWG – gestational weight gain

* p≤0.05; ** p≤0.01; *** p≤0.001

Supplement Captions:

Supplement Figure 1. Study flowchart

Supplement Figure 2A. Predicted probabilities derived from Model 2 (Genetic), Model 3 (Genetic+ Socio-demographic), and Model 4 (Genetic+ Socio-demographic+ Perinatal), separately for subjects with obesity (Panel A, C and E), subjects without obesity (Panel B, D and F)

Supplement Figure 2B. Predicted probabilities derived from Model 2 (Genetic), Model 3 (Genetic+ Socio-demographic), and Model 4 (Genetic+ Socio-demographic+ Perinatal), separately for subjects with high central obesity (Panel A, C and E) and without (Panel B, D and F).

Supplement Figure 1

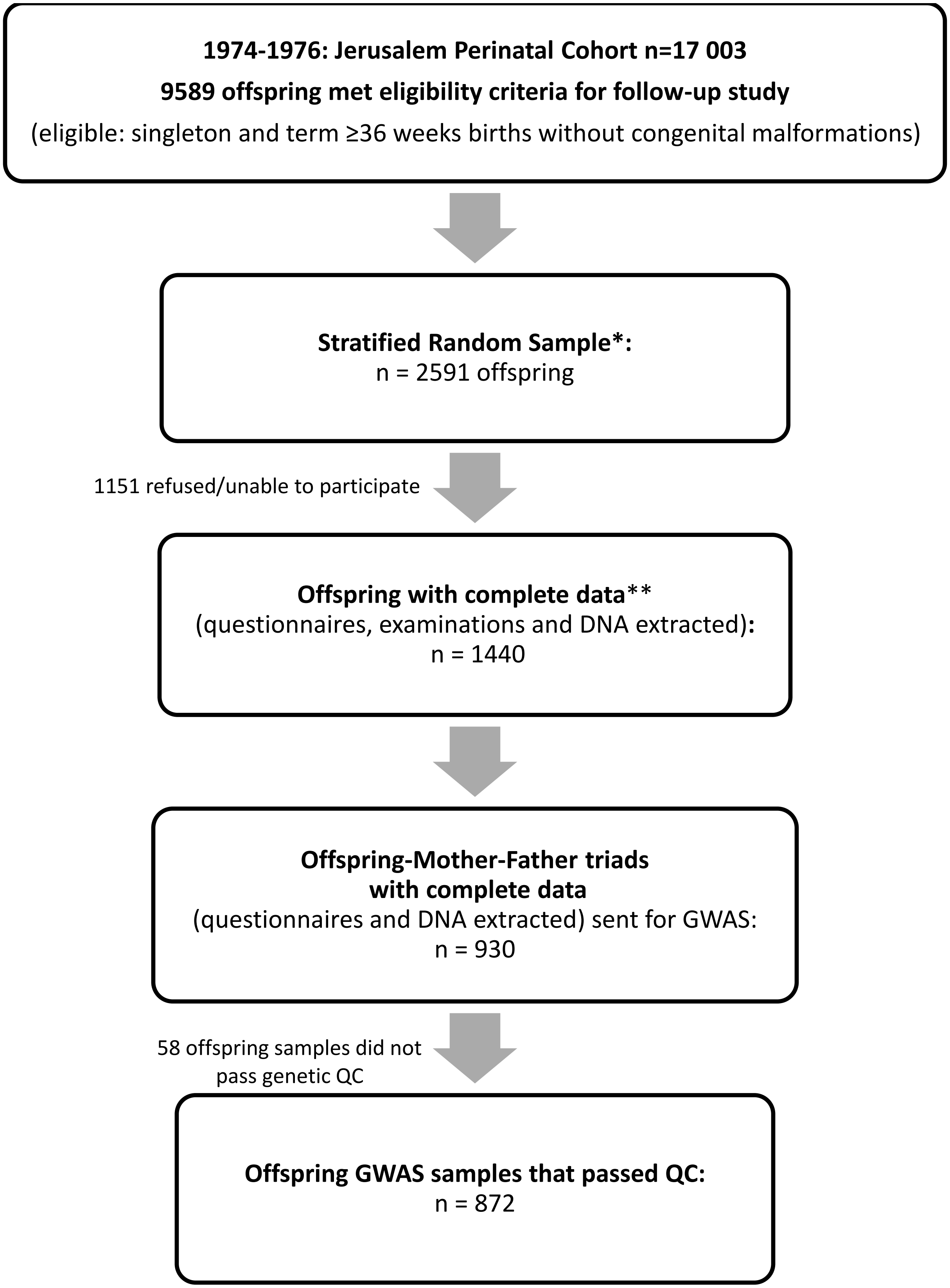

*strata defined by maternal pre-pregnancy BMI and birth weight where both low (≤2500 grams) and high (≥4000 grams) birth weight as well as overweight and obese mothers (BMI≥27) were over-sampled.

** The study sample was determined to provide an adequate power for study's aims.

Supplement Figure 2A

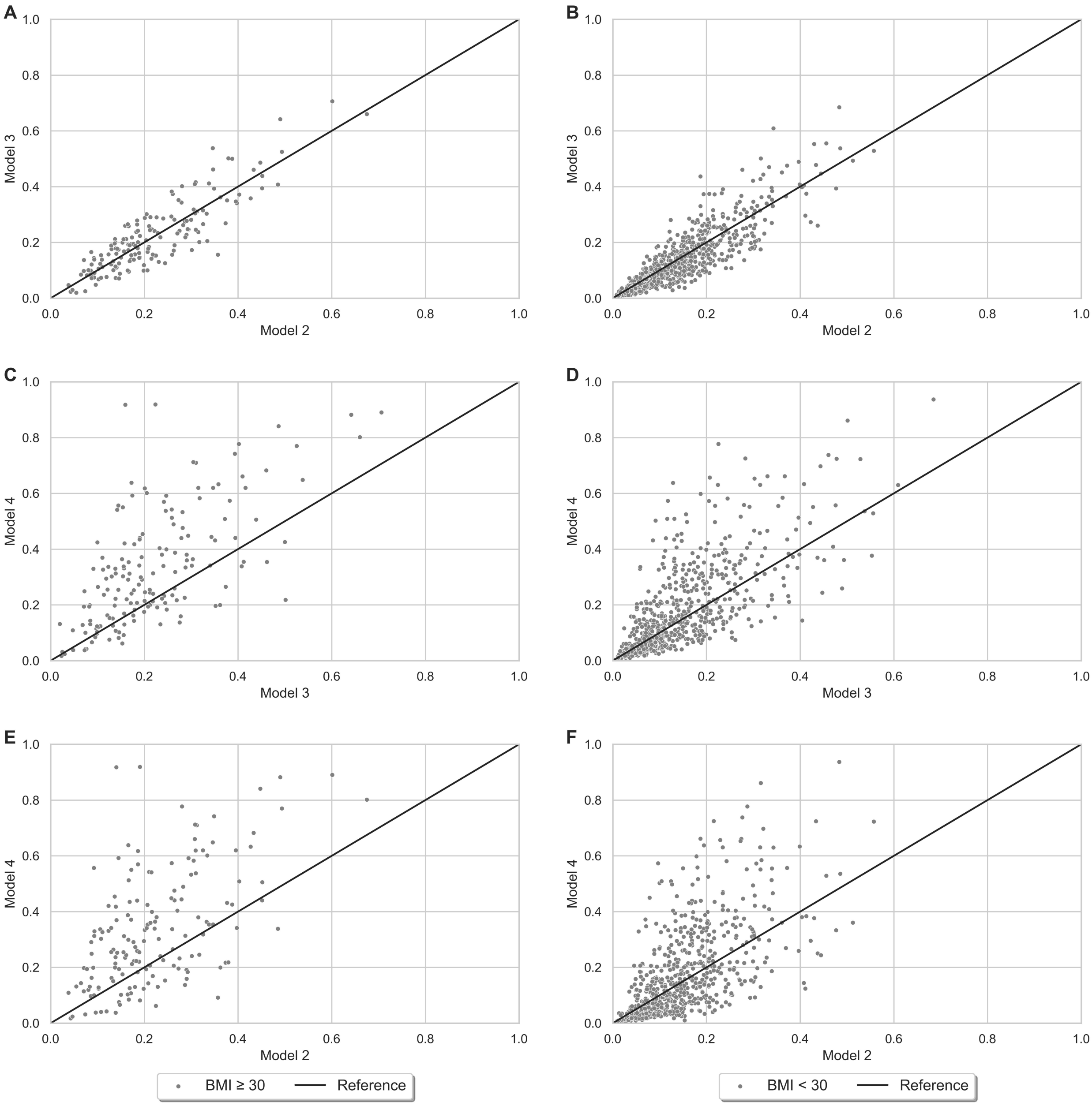

Supplement Figure 2B

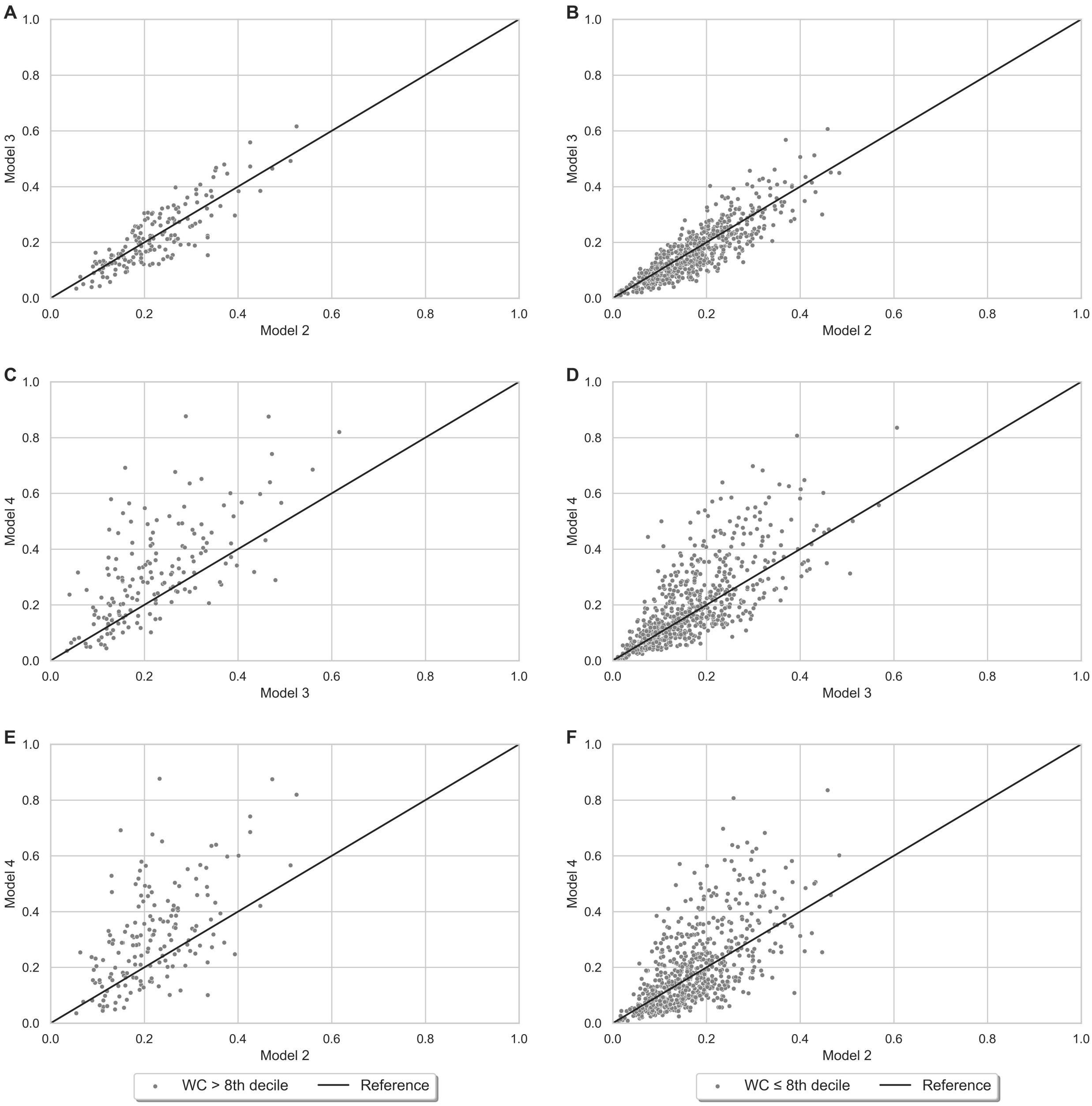

Supplement: Supplementary file 1 — Supplementary materials [file 41366_2024_1505_MOESM1_ESM.pdf]
